# Surgical management of periocular squamous cell carcinoma: case report

**DOI:** 10.25122/jml-2023-0537

**Published:** 2023-10

**Authors:** Anisia-Iuliana Alexa, Carmen-Ecaterina Leferman, Alin Dumitru Ciubotaru, Alexandra Maștaleru, Irina Mihaela Abdulan, Maria Magdalena Leon

**Affiliations:** 1 Department of Ophthalmology, “Grigore T. Popa” University of Medicine and Pharmacy, Iași, Romania; 2 Department of Pharmacology, “Grigore T. Popa” University of Medicine and Pharmacy, Iași, Romania; 3 Department of Neurology, “Grigore T. Popa” University of Medicine and Pharmacy, Iași, Romania; 4 Department of Medical Specialties I, “Grigore T. Popa” University of Medicine and Pharmacy, Iaşi, Romania

**Keywords:** squamous cell carcinoma, periocular skin tumors, oculoplastics, keratoacanthoma

## Abstract

This report describes the case of a 72-year-old female patient admitted to the ophthalmology clinic for a large round-oval tumor with a long-standing keratotic lesion on her lower eyelid, without extending to the free margin of the eyelid. The tumor was excised with a margin in non-tumorous tissue, the nearest being 1 mm away from the tumor at the 12 o’clock position. The surgical process was complicated by the patient’s treatment with the anticoagulant rivaroxaban, resulting in increased bleeding during surgery. The histopathological evaluation showed characteristics indicative of a well-differentiated squamous cell carcinoma, more specifically, the keratoacanthoma type. Consequently, it was necessary to extend the excision at the 12 o’clock position by an additional 3 mm. The procedure involved extensive removal of the impacted area and subsequent reconstruction with advancement flaps, supported by histological examination to ensure total excision. In cases of squamous cell carcinoma on the eyelid, multiple sequential excisions are often required to ensure complete removal within safe histological margins, achieving desirable functional and esthetic results.

## INTRODUCTION

In ocular oncology, periocular squamous cell carcinoma (SCC) is a notably aggressive neoplasm, accounting for a significant proportion of eyelid malignancies [1]. Specifically, it represents between 5% and 10% of all eyelid skin cancers, while constituting less than 2% of overall eyelid lesions [2]. Therefore, it is critical to better understand its pathogenesis and management strategies.

Cutaneous carcinomas of the periocular region have several histopathological subtypes (e.g., adenoid/acantholytic, mucin-producing, and verrucous forms), each presenting unique challenges and implications for treatment [3]. Because of their diversity, a tailored approach to diagnosis and therapy is necessary. In this context, an accurate histopathological assessment is pivotal.

The prognosis of periocular SCC is intricately linked to multiple factors, such as the anatomical location of the tumor, its histological characteristics, the underlying etiological factors, and its clinical features [3]. This dependency on several prognostic factors highlights the complexity of managing this condition and the importance of a comprehensive, tailored patient evaluation to optimize outcomes.

In this context, we report the case of a 72-year-old female patient with various chronic conditions and on multiple medications, who presented to the ophthalmology clinic with a massive, growing, keratotic tumor on her lower eyelid.

## CASE PRESENTATION

A 72-year-old female patient visited the ophthalmology clinic with a complaint of a large, progressively growing tumor on her lower eyelid. The tumor had a round-oval shape with keratinized extensions, and it has been developing gradually over several years without impacting the free margin of the eyelid. Its progressive nature and distinct morphological features raised clinical concerns, requiring a detailed assessment in the context of her overall health status.

Her extensive medical history revealed a variety of underlying conditions, such as permanent atrial fibrillation and New York Heart Association (NYHA) class II heart failure. Additionally, she presented grade 2 hypertension, minimal mitral and aortic insufficiency, and posterior mitral ring calcifications. Her health background was further complicated by neurological and psychiatric conditions like vestibular and depressive syndrome, as well as musculoskeletal issues, such as cervical spondylosis, and mixed dyslipidemia.

Her medical treatment was extensive and included carvedilol for cardiac support, the diuretics furosemide and spironolactone, the anticoagulant rivaroxaban, and the antiplatelet agent clopidogrel. Additionally, her psychiatric symptoms were treated with mirtazapine and nitrazepam.

### Ophthalmological examination

Ophthalmological examination revealed normal visual acuity in both eyes and normal intraocular pressure, with no nystagmus or abnormal eye movements. Both pupils were equal, round, and reacted to light and accommodation appropriately.

The slit lamp exam of the anterior segment was normal, with the exception of cortical crystalline opacities. Fundoscopic examination revealed normal results.

**Figure 1:**
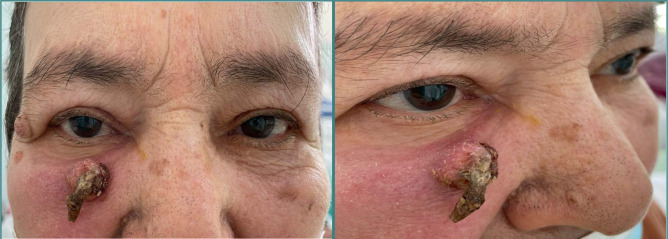
Keratotictumor located in the right lower edge of the periorbital area

Eyelid examination showed a round-oval formation of approximately 1 cm in diameter with keratinized extensions, adherent to the underlying tissues, located in the lower edge of the periorbital area (Figure 1).

### Differential diagnosis

When evaluating a hyperkeratotic lesion on the lower eyelid, a differential diagnosis is essential to distinguish among various potential etiologies. One primary consideration is actinic keratosis, which appears as a scaly or crusty bump arising on sun-exposed areas of the skin, including the eyelids. These lesions result from long-term exposure to ultraviolet light and carry a risk of progression to SCC. Another important differential diagnosis can be made with basal cell carcinoma or melanoma. This involves differentiating the scaly, often crusty lesions of SCC from the pearly, telangiectatic nodules of basal cell carcinoma, and the varied, often asymmetrical pigmentation with irregular borders characteristic of melanoma. SCC itself is another differential diagnosis, presenting typically as a thickened, red, scaly lesion that may ulcerate and invade surrounding tissues. Both conditions require histological examination to confirm the diagnosis and to guide treatment.

Another possibility includes seborrheic keratosis, which is a benign skin growth that often appears in older adults. These lesions are characterized by a waxy, stuck-on appearance and can vary in color. While typically asymptomatic, they can become irritated or cosmetically undesirable. Additionally, viral warts caused by human papillomavirus can present as hyperkeratotic lesions and should be considered, especially if there is a history of similar lesions elsewhere on the body or evidence of immunosuppression. Each of these conditions has distinct clinical features and treatment approaches and, at the same time, a different prognosis, necessitating a thorough evaluation, including patient history, physical examination, and possibly biopsy, to achieve an accurate diagnosis.

### Treatment

**Figure 2:**
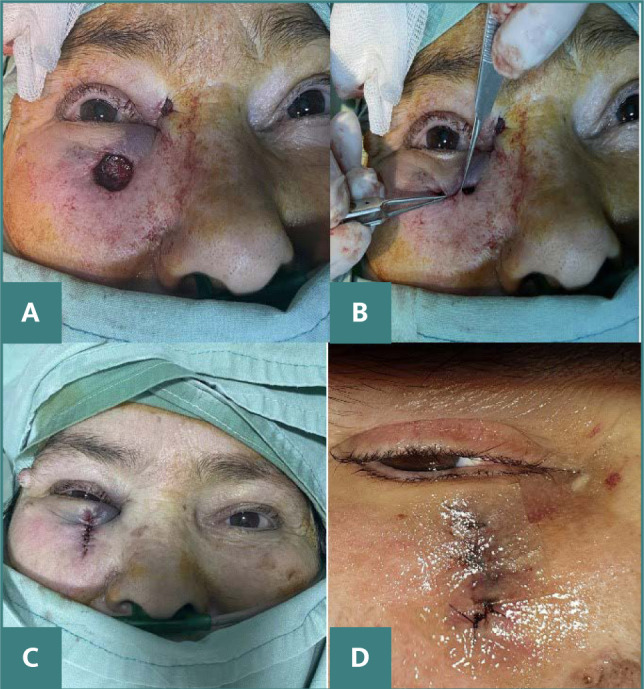
Intraoperative images A. The defect that remained following the complete excision of the keratotictumor. B. Manipulating the edge of the wound to facilitate closure of the defect, which was expanded by an additional 3 mm at the 12 o'clock position. C. Closing the defect with an advancement flap. D. Postoperative images at 10 days demonstrating satisfactory esthetic and functional results.

The patient underwent a two-step procedure for surgical excision of the tumor, where the margins of the removed tissue extended 4 mm into the surrounding clinically healthy tissue (Figure 2). This approach is intended to ensure that the entirety of the tumor is excised, reducing the likelihood of residual malignant cells and minimizing the potential for recurrence.

The histopathological examination of the first excised tissue revealed the margins were clear of tumor involvement in all planes, with the closest margin being 1 mm from the tumor at the 12 o’clock position. However, to ensure the thoroughness of the tumor removal, the excision was supplemented by an additional 3 mm at the 12 o'clock position (Figure 2). Following the confirmation through the second histopathological analysis that the excision was complete and the margins were within safe histological limits, the site underwent reconstruction using advancement flaps. This technique involved stretching adjacent healthy tissue over the excision site to facilitate healing and maintain functional integrity, and to ensure optimal esthetic and functional outcomes (Figure 2).

The diagnosis confirmed by the histopathological examination was of a well-differentiated keratoacanthoma type SCC of the eyelid. Histologically, keratoacanthoma-type SCC is characterized by well-differentiated squamous cells. It may also exhibit a cup-shaped architecture with a central area of keratinization, which is typical of keratoacanthomas. Clinically, it may be difficult to distinguish from benign keratoacanthomas, which are usually self-limiting; hence, a biopsy is needed for accurate diagnosis.

### Outcome

There were no complications during postoperative follow-up, and six months after discharge the patient presented good functional and esthetic results.

## DISCUSSION

In the presented case, the clinical diagnosis of the keratotic lesion highlights the clinical and histological similarities between keratoacanthoma and periocular SCC [4]. The main difference between them is the potential for spontaneous regression in the case of keratoacanthoma. However, some authors consider keratoacanthoma a variant of SCC, which is supported by the lack of particularly different immunohistochemical findings between keratoacanthoma and well-differentiated SCC [4].

An imprecise clinical diagnosis makes the treatment of keratotic lesions difficult. Options include observation, surgical excision with histological control of the excision margins, radiotherapy, and cryotherapy [5, 6]. Although keratoacanthoma can regress spontaneously, periocular SCC has an aggressive malignant potential, presenting possible local recurrence [6]. These are influenced by treatment, location, depth of the tumor, histological differentiation, perineural involvement, precipitating factors other than ultraviolet light, host immunosuppression, and desmoplastic changes [7, 8]. Therefore, an excisional biopsy of keratotic eyelid lesions is recommended rather than observation, to see if the lesions regress [9]. In the presented case, the lesion was large; therefore, the removal of the formation required an additional excision.

Because of the anatomical peculiarities of the eyelids, serial excisions are sometimes necessary to achieve favorable esthetic and functional surgical outcomes [10]. In the case of this patient, in order to obtain excision margins of 4 mm, as indicated in the literature [11], a supplementary intervention was necessary. Moreover, as the patient was under chronic anticoagulant treatment, the difficulty of the surgical intervention was increased by significant intraoperative bleeding. Also, the surgical defect resulting after the complete excision within safe histological limits indicated reconstruction with advancement flaps as the best therapeutic option. Another possible treatment worth mentioning, with certain financial and logistical disadvantages, is the Mohs technique, which also aims to completely remove skin cancer while minimizing damage to the surrounding healthy tissue [12].

## CONCLUSION

The presented case highlights the challenges in achieving an accurate clinical diagnosis of SCC and proves the efficacy of a definitive treatment protocol involving surgical excision of all lesions suspected as SCC. In this regard, because of the anatomical particularities of the eyelids, serial excisions may be needed to achieve complete removal within safe histopathological margins, with good esthetic and functional outcomes.

## Data Availability

Further data are available from the corresponding author upon reasonable request.
